# Proline-directed phosphorylation and prolyl isomerization oppose each other to regulate PSD-95 ubiquitination and excitatory synaptic plasticity

**DOI:** 10.3389/fnmol.2026.1777680

**Published:** 2026-06-16

**Authors:** Prajwal Kurup, Abigail R. Thielbar, Evan Jasinski, Briahna Galvan, Taran Singh, Sai Kanuru, Het T. Gor, Adalia Von Rommel, Maria R. Parungao, Daniel B. Paiva Parada, Meera J. Patel, Jary Y. Delgado

**Affiliations:** Department of Biology, Loyola University Chicago, Chicago, IL, United States

**Keywords:** GSK3β, NMDAR-LTD, phosphorylation, Pin1, PSD-95, synaptic plasticity, ubiquitination

## Abstract

PSD-95 is a key scaffolding protein in excitatory synapses, playing a critical role in stabilizing synaptic receptors. Post-translational modifications such as phosphorylation and ubiquitination have been shown to regulate PSD-95’s localization and stability. Threonine 19 (T19) phosphorylation has emerged as a key phosphorylation site involved in NMDAR-dependent LTD; however, its role in PSD-95 ubiquitination is unknown. This study aims to elucidate the role of T19 phosphorylation in regulating PSD-95 ubiquitination, and whether the peptidyl prolyl isomerase 1 (Pin1), a known phospho-T19 binding protein, modifies this process. Our results support the idea that T19 and S25 phosphorylation positively regulate PSD-95 ubiquitination. Furthermore, we found that the Pin1 binding to S25 lowered the phosphorylation state of T19 and prevented PSD-95 ubiquitination. Finally, whole-cell recordings show that GSK3 overexpression depressed baseline synaptic transmission and partially blocked NMDAR-LTD, and both effects were rescued by Pin1 overexpression. Together, these findings provide insights into the molecular mechanisms regulating PSD-95 stability at synapses.

## Introduction

Synaptic plasticity, the ability of synapses to strengthen or weaken over time, is critical for learning and memory processes. Long-term depression (LTD) is a form of synaptic plasticity that involves the weakening of synaptic connections and is known to depend on N-methyl-D-aspartate receptor (NMDAR) activation. A key player in the regulation of synaptic plasticity is the postsynaptic density protein-95 (PSD-95), a scaffolding protein that anchors glutamate receptors at excitatory synapses. The stability of PSD-95 at synapses is dynamically regulated by post-translational modifications, including phosphorylation, palmitoylation, and ubiquitination (Bianchetta et al., 2011; Ho et al., 2011; Morabito et al., 2004; Nelson et al., 2013; Topinka and Bredt, 1998; Zhu et al., 2013), which collectively influence synaptic composition and strength.

The N-terminus domain of PSD-95 contains two well-characterized phosphorylation residues, threonine 19 (T19) and serine 25 (S25) (Bianchetta et al., 2011; Morabito et al., 2004). Phosphorylation of T19 by glycogen synthase kinase-3β (GSK3β) has been implicated in NMDAR-dependent LTD (Nelson et al., 2013), whereas S25 is phosphorylated by cyclin-dependent kinase 5 (CDK5) and has been linked to activity-dependent destabilization of PSD-95 (Bianchetta et al., 2011). While recent studies have highlighted a prominent role for GSK3*α* in NMDAR-dependent LTD in CA1 pyramidal neurons (Draffin et al., 2021), the extent to which individual GSK3 isoforms regulate postsynaptic scaffold stability remains unclear. While phosphorylation of the PSD-95 N-terminus is clearly involved in LTD, how these phosphorylation events intersect with ubiquitination—an essential mechanism controlling PSD-95 turnover and the α-amino-3-hydroxy-5-methyl-4-isoxazolepropionic acid receptor (AMPAR)—remains incompletely understood.

PSD-95 ubiquitination is mediated by the E3 ubiquitin ligase MDM2 (Bianchetta et al., 2011; Colledge et al., 2003); yet studies differ on whether PSD-95 undergoes mono- or poly-ubiquitination and on the functional consequences of each modification (Bianchetta et al., 2011; Bingol and Schuman, 2004; Ma et al., 2017). The level of mono- and poly-ubiquitination of PSD-95 is sensitive to the activity levels of CDK5, with these levels increasing in the p35 knockout mice (Bianchetta et al., 2011). The ubiquitination of PSD-95 can take place in various lysine residues distributed throughout the protein (Bianchetta et al., 2011), including lysine 10, which lies in close proximity to T19 and S25. Notably, phosphorylation of S25 has been shown to inversely correlate with PSD-95 monoubiquitination during chemically induced LTD, promoting AMPAR endocytosis through *β*-adaptin–dependent pathways (Bianchetta et al., 2011). These observations suggest a regulatory relationship between N-terminal phosphorylation and PSD-95 ubiquitination; however, whether phosphorylation at T19 similarly regulates PSD-95 ubiquitination has not been directly evaluated.

In addition to kinase-dependent phosphorylation, PSD-95 stability is regulated by the phosphorylation-dependent prolyl isomerase Pin1, which binds phosphorylated Ser/Thr-Pro motifs and catalyzes cis–trans isomerization (Delgado, 2020). Pin1 preferentially associates with phosphorylated T19 over S25 in the PSD-95 N-terminus and modulates PSD-95 palmitoylation, leading to changes in synapse number and stability (Delgado et al., 2020). Although Pin1 has been shown to regulate ubiquitination of multiple non-synaptic proteins (Siepe and Jentsch, 2009; Liou et al., 2011; Nakano et al., 2009; Zhou et al., 2009), its role in the ubiquitination of synaptic scaffolding proteins has not been examined.

Here, we investigated how phosphorylation at T19 and S25 regulates PSD-95 ubiquitination during NMDAR-dependent LTD and examined the role of Pin1 in modulating this process. Using a combination of biochemical approaches and single-cell electrophysiology, we demonstrate that phosphorylation at both T19 and S25 regulates PSD-95 ubiquitination, identify Pin1 as a negative regulator of this modification, and show that opposing actions of Pin1 and GSK3β converge to regulate synaptic transmission and LTD.

## Materials and methods

### HEK 293 T cell culture

HEK 293 T cells were plated in DMEM media supplemented with 10% FBS and 1X Pen/Strep as described in our previous publication (Delgado, 2020). Cells were plated onto 100 mm plates at a confluence of 10,000,000 cells per plate. Four to six hours post plating, cells were then transfected using the calcium phosphate transfection method with plasmids expressing HA-Ubiquitin (pRK5-HA-Ubiquitin-WT, ID: 17608, Addgene), PSD-95, HA-GSK3β (HA GSK3 beta wt pcDNA3, ID: 14753, Addgene), and Pin1 (Delgado et al., 2020). A total of 10 μg total cDNA was used per plate. Cells were then placed back in the incubator and left to grow. Cells were fed by replacing the media 24 h post transfection. Cells were later harvested, 48 h post transfection, by scraping them off in 1X PBS at 4 °C. Samples were then spun at 800 x g for 5 min at 4 °C in a tabletop centrifuge to separate the cell pellet. The resulting supernatant was aspirated, and the pellets were immediately frozen at −80 °C.

### SDS-PAGE electrophoresis and western blotting

Protein samples were prepared from the frozen pellets. Cells were lysed in a buffer containing 50 mM Tris–HCl, 200 mM NaCl, 100 mM NaF, 10% Glycerol, 1% Triton X-100, and protease inhibitor cocktail III (Calbiochem) to pH 8. The nuclear and mitochondrial fraction was removed by centrifugation at 800 x g for 10 min at 4 °C. The resulting pellet is sonicated briefly (for 3 s at 4 °C) on an ultrasonic homogenizer and the resuspended solution is spun once more. The resulting supernatant was collected, and the protein concentration quantified using the BCA method.

For regular western blots, equal amounts of protein were mixed with 4x Laemmli buffer, diluted to 1X, and boiled at 95 °C for 6 min. Proteins were separated on 10% SDS-polyacrylamide gels at 120 V for approximately 90 min in running buffer (25 mM Tris, 19 mM Glycine, 0.1% SDS). Following electrophoresis, the proteins were transferred to PVDF membranes using a wet transfer system at 100 V for 1 h on ice (Bio-Rad Mini Trans-Blot^®^ Cell and Criterion^™^ Blotter). The membranes were blocked in 5% non-fat milk in TBST (Tris-buffered saline with 0.1% Tween-20) for 1 h at room temperature. Primary antibodies against PSD-95 (K28/43, at a dilution of 1:400, from cell supernatant), HA tagged proteins (GSK3β and HA-ubiquitin, 12CA5, at a dilution of 1:5,000), and Pin1 (G-8, at a dilution of 1:4,000) were diluted in 4% non-fat milk in TBST and incubated overnight at 4 °C in a rocker. For the phospho-T19 experiment, membranes were blocked in 4% BSA. The next day, membranes were washed three times in TBST and incubated in 4% non-fat milk in TBST with Southern Biotech HRP-conjugated secondary antibodies at a 1:10,000 dilution for 2 h at room temperature. Protein bands were visualized using enhanced chemiluminescence (ECL) and detected using a chemiluminescence imaging system (Odyssey^®^ XF Imaging system LICORbio^™^). Images were imported to FIJI and the intensity of the protein bands were quantified using the gel analyzer function. Antibodies were then stripped using a mild stripping buffer (200 mM Glycine, 0.1% SDS, 1% Tween 20) as follows; the membrane was washed twice with a mild stripping buffer for 10 min each, followed by a 10-min wash with PBS. Three washes with TBST for 5 min each were performed. The membrane was blocked for one hour with 4% non-fat milk in TBST to prevent nonspecific protein binding. The ratios of ubiquitinated PSD-95 to total PSD-95 were calculated for statistical comparison.

For immunoprecipitation experiments, HEK293-T cells were transfected and lysed as described before. Approximately, two hundred micrograms of total protein (depending on cell confluence) were incubated with 2 μg of anti-PSD-95 antibody (K28/43) or anti-MDM2 antibody (SMP14) for a minimum of 1 h followed by 2 h incubation with 15 μL of Cytiva Protein G Mag Sepharose^™^ (Fisher) pre-blocked with 1% BSA. Protein complexes were thoroughly washed three times with 200 μL of ice-cold lysis buffer and eluted in a new tube using 50 μL of 1X Laemmli buffer and run in an SDS-PAGE gel.

### Electrophysiology

Pooled cultured rat hippocampal neurons from both male and female rat embryos were used. Hippocampal neurons were cultured from E18.5 rat embryos and grown in Neurobasal media supplemented with B27 supplement, 2.5% fetal bovine serum (FBS), and GlutaMax as described in Delgado et al. (2020) and Galvan et al. (2025). Neurons were plated onto 22 × 22 mm 1.5 glass cover slips. 250,000 to 400,000 neurons were plated per coverslip. 1 μM Ara-C was applied a day or two after. Cultured neurons were transfected 7 days post plating (using Lipofectamine 3,000 following the manufacturer’s recommendation) with 1 μg of pIRES2-DsRed-Express2 plasmids along with the following proteins of interest: Pin1 IRES2 EGFP, Pin1 shRNA, or with GSK3β. Following transfection, cultures were fed every 3 to 4 days and maintained for up to a month.

Whole-cell voltage-clamp experiments were conducted on 14 to 20 days *in vitro* (DIV) cultured neurons. Neurons were visualized using IR-DIC on a fixed stage upright Zeiss Examiner. A1 electrophysiology suite microscope and the identification of pyramidal neurons was achieved based on the red fluorescence coming from DsRed when excited with our RFP dichroic mirror. Recordings were performed at 30–32 °C in oxygenated artificial cerebrospinal fluid (ACSF) supplemented with 1 mM sodium pyruvate as in Delgado et al. (2020). Recording pipettes (3–6 MΩ) were filled with a cesium-gluconate or potassium-gluconate internal solution and the osmolality was adjusted to 288 mOsm using sucrose. Recording quality was assessed throughout the experiment (series resistance <20 MΩ, input resistance <30% variation). Cells demonstrating changes in these parameters were excluded from analysis. mEPSCs were isolated in the presence of 1 μM TTX, 20 μM bicuculine, and 50 μM APV. It is possible to record from these neurons for up to an hour.

Several LTD protocols were tried without much success. The chemical LTD approach was selected for its reliability. Chem LTD was induced as in Beattie et al. (2000) and Lee et al. (1998) with the following modifications. A whole-cell voltage-clamp was established and after the patch is stable, a 5-min recording was acquired, then 20 μM NMDA was applied to the coverslips for 5 min. The NMDA solution was delivered with modified concentrations of divalent ions (4 mM Ca^2+^/1.2 mM Mg^2+^) which we have determined to reliably induces LTD (Delgado et al., 2007). The arrival of NMDA was determined by the increase in holding current measured by the amplifier. At that time, the amplifier was quickly switched to current clamp. Neurons were left to be depolarized for the rest of the time. After the NMDA, the cells were left to recover the resting membrane potential as in Lee et al. (1998), and once the resting potential was reestablished the amplifier was switched back to voltage clamp to record mEPSCs for another 20 to 30 min. Some cells did not survive the protocol and were discarded.

### Experimental design and statistical

Data collection was interleaved, to control for time and order effects. Samples size was determined using a power analysis calculator based on the known variability of a sample experiment. For each experiment, data from all groups were acquired weekly to reduce variability or else it was not included in the analysis. For electrophysiology an un-paired, paired or One-Way ANOVAs statistical analysis design was employed. For biochemistry, un-paired and One-Way ANOVAs were utilized. Normality testing was performed on every group using D’Agostino and Pearson omnibus normality tests. Between- group statistical significance was calculated accordingly for each distribution and experiment design. Data were normalized on a weekly basis to compensate for week-to-week variability. Numerical averages are presented as mean ± SEM or as box plots. Statistical analyses were created using Excel or using R. Exact *p*-values are reported when provided. At the end of a dataset, the groups were tested for the presence of outliers using the Prism online calculator at a significance level of *p* < 0.05. Only then were these eliminated.

## Results

### GSK3β regulates PSD-95 ubiquitination

The N-terminus domain of PSD-95 contains two well-characterized proline-directed phosphorylation residues, threonine 19 (T19) and serine 25 (S25) (Bianchetta et al., 2011; Coba et al., 2009; Morabito et al., 2004). The third proline-directed phosphorylation site, S35, has yet to be studied systematically. T19 has been shown to be the target of GSK3β phosphorylation during NMDAR-dependent LTD (Nelson et al., 2013) and S25 has been shown to be phosphorylated by CDK5 (Morabito et al., 2004). While the phosphorylation of the N-terminus domain of PSD-95 has been well established, its specific impact on PSD-95 ubiquitination has not been explored. To further investigate this relationship, the following experiments examine how changes in T19 alters its ubiquitination.

**Figure 1:**
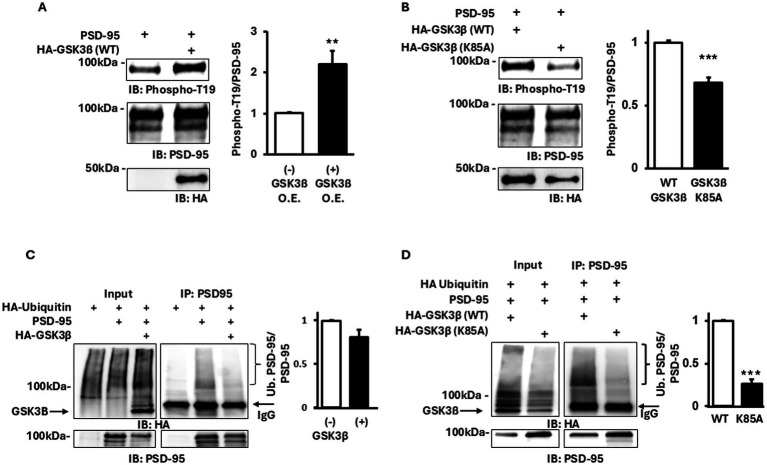
Effects of GSK3β on PSD-95 ubiquitination. **(A)** Representative immunoblot and accompanying graph demonstrating the increase in phosphorylated PSD-95 levels in the presence of the GSK3ß overexpression. (−) GSK3ß 1.02 ± 0.02; (+) GSK3ß 2.16 ± 0.34, *n* = 9, **p = 0.003, un-paired t-test. **(B)** Representative immunoblot and accompanying graph demonstrating the increase in phosphorylated PSD-95 levels in the presence of the GSK3ß overexpression vs. in the presence of the K85A kinase dead mutant of GSK3B. GSK3β 1 ± 0.011, *n* = 6; K85A GSK3β 0.67 ± 0.085, *n* = 6, un-paired t-test, ***p = 0.0051. **(C)** Representative immunoblot showing the immunoprecipitation of ubiquitinated PSD-95 in cells overexpressing wt GSK3B. Levels of ubiquitinated proteins measured for the signals greater than 100 kDa. (Right) quantification of the HA signal normalized to the signal for total PSD-95. (−) GSK3β 0.99 ± 0.005, *n* = 6; (+) GSK3β 0.808 ± 0.082, *n* = 6, un-paired t-test, *p* = 0.069. **(D)** Representative immunoblot showing the immunoprecipitation of ubiquitinated PSD-95 in cells overexpressing wt GSK3B or the K85A mutant. (right) Quantification of the HA signal normalized to the signal for total PSD-95 showing a statistically significant decrease in the levels of PSD-95 ubiqutination, wt GSK3β 1 ± 0.013, *n* = 8; K85A GSK3β 0.26 ± 0.052, *n* = 8, un-paired t-test, ***p < 0.001. Statistical significance is denoted by an asterisk. Results suggest a strong correlation between K85A overexpression and decreased levels of ubiquitinated PSD-95.

To confirm that T19 is a target of GSK3β phosphorylation HEK293T cells were cotransfected with plasmids encoding PSD-95 and GSK3β. Lysates overexpressing GSK3β displayed a significant increase in the phospho-T19 signal compared to control conditions (Figure 1A, (−) GSK3β 1 ± 0.02, *n* = 9; (+) GSK3β 2.16 ± 0.34, *n* = 9, un-paired t-test, *p* = 0.00305), confirming GSK3β-mediated phosphorylation of PSD-95 at T19 (Nelson et al., 2013). While overexpressing the kinase dead form of GSKβ reduced the phosphorylation level measured by the phospho-T19 antibody (Figure 1B, wt GSK3β 1 ± 0.011, *n* = 6; K85A GSK3β 0.67 ± 0.085, *n* = 6, un-paired t-test, *p* = 0.0051), suggesting that T19 phosphorylation can be maintained under basal conditions and is sensitive to manipulations of kinase activity.

Having confirmed that T19 phosphorylation is sensitive to kinase activity, we investigated if increases in T19 phosphorylation regulates PSD-95 ubiquitination. HEK293T cells were cotransfected with plasmids encoding HA tagged ubiquitin, PSD-95 and GSK3β. PSD-95 was immunoprecipitated (IP) and the amount of ubiquitinated PSD-95 was revealed with an antibody recognizing the HA tag. As expected, a strong HA signal was observed in the input lanes for all conditions (Figure 1C). IP from cells not expressing PSD-95 failed to show a signal corresponding to the mono, multi-monoubiquitnated, and polyubiquitinated forms of PSD-95 (Figure 1C, 1st lane right panel), overexpressing PSD-95 show a pronounced band migrating above the 100 kDa to approximately 116 kDa (Figure 1C, 2nd lane right panel), possibly the product multi-monoubiquitinated forms of PSD-95 as shown previously (Bianchetta et al., 2011). Overexpressing GSK3β (+ lane) did not increase or decrease the levels of PSD-95 ubiquitination (Figure 1C, (−) no GSK3β being overexpressed 0.99 ± 0.005, *n* = 6; (+) GSK3β overexpression 0.808 ± 0.082, *n* = 6, un-paired t-test, *p* = 0.069), suggesting that this ubiquitination of PSD-95 is under additional control. To further test the regulation of PSD-95 ubiquitination by phosphorylation, we repeated the IP protocol from cells HEK293T cells were cotransfected with plasmids encoding HA tagged ubiquitin, PSD-95 and wt GSK3β or the kinase dead GSK3β K85A mutant. We used the K85A construct to selectively interfere with endogenous GSK3β function. This approach provides specific inhibition at the level of substrate interaction while avoiding potential off-target effects of inhibitory drugs, and compensatory signaling changes associated with pharmacological inhibition. Genetic inhibition also ensures that kinase suppression occurs specifically in transfected cells, allowing direct comparison with control conditions. PSD-95 ubiquitination was clearly measurable in cells overexpressing wt GSK3β, but in cells expressing the kinase dead GSK3β K85A mutant there was a marked decrease in PSD-95 ubiquitination (Figure 1D, wt GSK3β 1 ± 0.013, *n* = 8; K85A GSK3β 0.26 ± 0.052, *n* = 8, un-paired t-test, *p* < 0.001), which is similar in direction, but not in magnitude, to the statistically significant reduction in phosphorylation at T19 observed in cells overexpressing the kinase dead mutant (Figure 1B), indicating that loss of kinase activity reduces both phosphorylation and ubiquitination, although the larger reduction in ubiquitination suggests additional mechanisms beyond phosphorylation alone may contribute to its regulation. These findings support the idea that baseline phosphorylation of T19 plays a key role in promoting PSD-95 ubiquitination, highlighting a potential mechanistic link between these two post-translational modifications.

### Regulation of PSD-95 ubiquitination by phosphorylation of the N-terminus of PSD-95 by GSK3β and Pin1

The N-terminus domain of PSD-95 contains two proline-directed phosphorylation sites (T19 and S25), with S35 also being subject to phosphorylation, and three other proline-directed phosphorylation sites within the hinge domain of the protein (Coba et al., 2009; Delgado, 2020; Delgado et al., 2020; Morabito et al., 2004). In particular, the N-terminus PEST domain in PSD-95 has been shown to be subjected to proteolytic degradation and to be required for ubiquitination, specifically at lysine 10 (Colledge et al., 2003; Lu et al., 2000; Xu et al., 2008). Furthermore, our findings shown in Figure 1 demonstrate that kinase activity regulate PSD-95 ubiquitination; however, whether these phosphorylation sites regulates PSD-95 ubiquitination is unknown.

**Figure 2:**
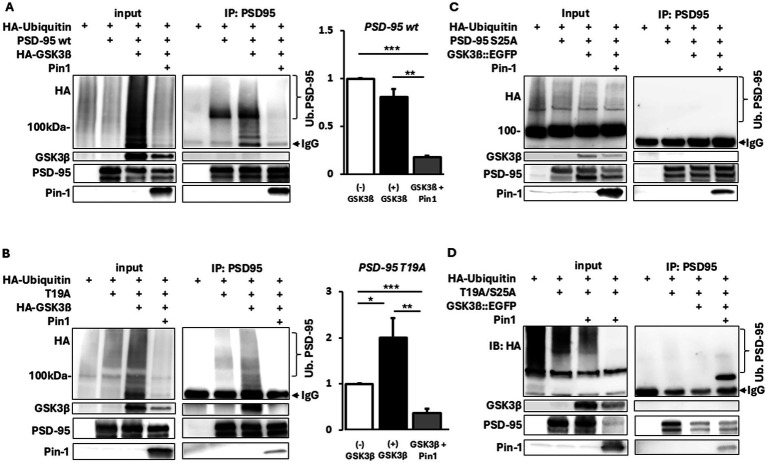
The phosphorylation of the N-terminus domain in PSD-95 regulates its ubiquitination state. Western blot showing the ubiquitination of wt PSD-95 **(A)**, and its mutants **(B–D)** in the presence of GSK3ß (3rd lanes) and Pin1 (4th lanes). **(A–D)** Representative western blot for input (left blot) and the immunoprecipation (right blot) for wt, T19A, S25A, and the T19A/S25A double mutantPSD-95, respectively. For wt PSD-95 **(A)** and the T19A mutant **(B)** there is ubiquitination in the lanes containing GSK3ß. Ubiquitination is absent in the presence of Pin1. Quantification of the respective data is shown in the bar graph next to the gel. **(C,D)** For the T19A **(C)** and the T19A/S25A double mutant **(D)** there is absence of ubiquitination in the lanes containing GSK3ß. Adding Pin1 does not reduce it further. No quantification is shown as there was no signal to measure.

To examine whether T19 and S25 phosphorylation regulate PSD-95 ubiquitination, we create phosphorylation-deficient forms of T19 and S25 by replacing those with alanine and compare the ubiquitination level to those in wt PSD-95. HEK293T cells were cotransfected with plasmids encoding HA tagged ubiquitin, the PSD-95 mutants and HA tagged GSK3β. PSD-95 was then immunoprecipitated. There was a reduced level of ubiquitination when compared to conditions expressing wild type PSD-95 (Figure 2B). (Figure 2A, (−) GSK3β 1.00 ± 0.005; (+) GSK3β 0.81 ± 0.08; (+) GSK3β & Pin1 0.18 ± 0.02). One-way ANOVA revealed a significant effect of condition, F(2,12) = 59.19, p = 6.08 × 10^−7^. Bonferroni-corrected post hoc comparisons showed significant differences between −GSK3β and +GSK3β + Pin1, as well as between +GSK3β and +GSK3β + Pin1 (all corrected **p < 0.001, ***p < 0.0001). In the T19A mutant (Figure 2B), overexpression of GSK3β led to a statistically significant increase in the levels of PSD-95 ubiquitination (Figure 2B, following a one-way ANOVA with Tukey’s comparison (−) GSK3β 1.00 ± 0.007; (+) GSK3β 1.99 ± 0.42, *n* = 10, F(2, 27) = 10.89, *p = 0.00034). Bonferroni-corrected post hoc comparisons showed significant differences between − GSK3β and + GSK3β + Pin1, as well as between + GSK3β and + GSK3β + Pin1 (all corrected *p < 0.05, **p < 0.01, ***p < 0.001). We next tested the role of S25 phosphorylation by transfecting the S25A mutant of PSD-95 instead. Under this condition, five out of five replicates exhibited no detectable signal (Figure 2C). Similarly, the double mutant of PSD-95 where T19 and S25 were mutated to alanine, failed to demonstrate any ubiquitination in 6 out of 9 replicates (Figure 2D). A brief note, GSK3β came down with the PSD-95 mutants containing the T19A mutation (9 out of 9 IPs) and T19A/S25A (3 out of 14 Ips), but it was not detectable in the wt IP lanes or the S25A alone. Together, this data suggests that ubiquitination is permitted when PSD-95 is phosphorylated at S25 and T19 playing a regulatory role or that additional sites are playing a role.

The regulation of PSD-95 ubiquitination by GSK3β in the T19 mutant appears to occur when GSK3β is associated with phosphorylated S25, but it could be that the T19A mutation exposes other regions in PSD-95 that become accessible for GSK3β association (Dajani et al., 2001; Plyte et al., 1992). Threonine 19 in PSD-95 is followed by proline 20 (T19-P), which we found to be a Pin1 binding site (Delgado, 2020; Delgado et al., 2020). Pin1 has been shown to regulate the level of protein ubiquitination in a substrate- and phosphorylation-specific manner (Hamdane et al., 2006; Kimura et al., 2013; Raghuram et al., 2013; Saxena et al., 2010; Tong et al., 2015; Zhou et al., 2000). In some cases, Pin1 favors ubiquitination and degradation of the phosphorylated target (Lonati et al., 2014; Nakano et al., 2009; Reineke et al., 2008; Schelhorn et al., 2015), while in other phosphorylated proteins, Pin1 favors deubiquitination and stabilization (Brondani et al., 2005; Nechama et al., 2009; Shen et al., 2005; Wulf et al., 2002; Zhou et al., 2009). Therefore, the role of Pin1 in regulating PSD-95 ubiquitination is unknown. To determine if Pin1 association modulates PSD-95 ubiquitination, we added Pin1 to the transfection mix (4th lane in Figure 2). While the PSD-95 ubiquitination was fairly robust, adding Pin1 to the transfection triggered a significant loss of ubiquitination in the input lanes (Figure 2, compare 3rd and 4th lanes for panels A – D) and a complete loss of ubiquitination in the IP condition (Figure 2A. To determine how the phosphorylation sites regulate Pin1-mediated ubiquitination of PSD-95, we performed the IP on cells transfected with the mutants of PSD-95. In lysates expressing the T19A mutant there was a complete loss of PSD-95 ubiquitination when Pin1 was overexpressed, suggesting that Pin1 could regulate PSD-95 ubiquitination via this site (Figure 2B, GSK3β 1.99 ± 0.42 vs. GSK3β + Pin1 0.36 ± 0.09, One-way ANOVA F(2, 27) = 10.89, *p* = 0.00034). Bonferroni-corrected post hoc comparisons showed significant differences between − GSK3β and + GSK3β + Pin1, as well as between + GSK3β and + GSK3β + Pin1 (all corrected *p < 0.05, **p < 0.01, ***p < 0.001). Similar effects were observed in cells expressing the S25A mutant. In this condition the effect was more dramatic because the ubiquitination was lost and adding Pin1 did not cause any further effects, suggesting that S25 is a primary site of action of Pin1 (Figure 2C). Lastly, the double mutant of PSD-95 was undistinguishable from the T19 or the S25 mutant, indicating that the effect of Pin1 on the ubiquitination of PSD-95 is through the regulation of these sites. Taken together, this data indicates that the phosphorylation of T19 and Pin1 association plays a determining role in regulating PSD-95 ubiquitination.

### Pin1 regulates the T19 phosphorylation over a slower time scale

**Figure 3:**
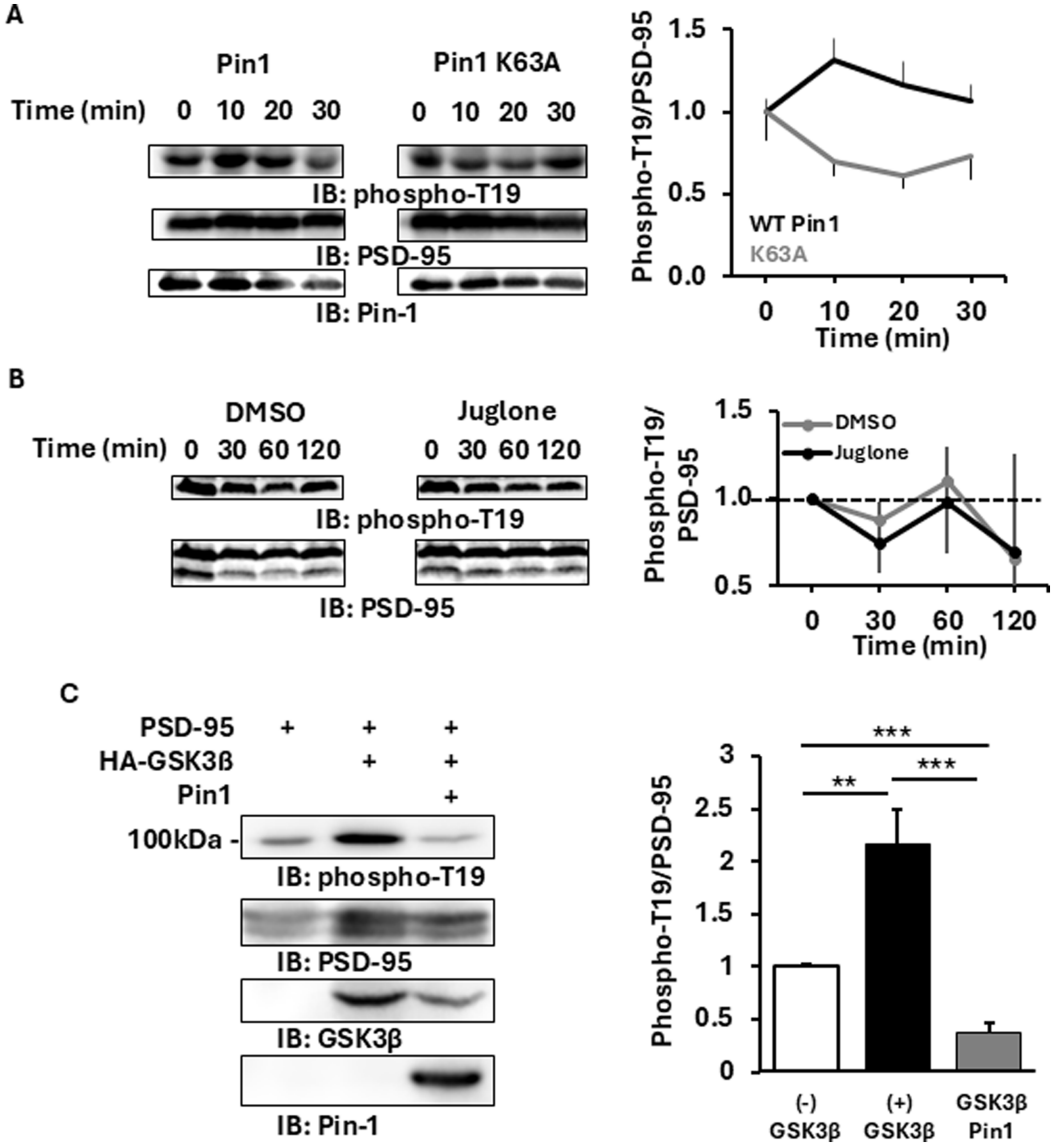
Pin1 regulates T19 phosphorylation of PSD-95 on a slow timescale. **(A)** Representative immunoblots and quantification of phospho-T19 levels in cells expressing wild-type Pin1 or the catalytically inactive mutant Pin1 K63A over a 30-min time course (0–30 min). Phospho-T19 levels were normalized to total PSD-95. Overexpression of wild-type Pin1 produced a modest increase in phospho-T19 levels, whereas expression of Pin1 K63A resulted in a reduction relative to baseline (Wt Pin1: T = 0, 1.00 ± 0.08; T = 10, 1.31 ± 0.13; T = 20, 1.17 ± 0.13; T = 30, 1.06 ± 0.10 vs. K63A: T = 0, 1.00 ± 0.17; T = 10, 0.70 ± 0.08; T = 20, 0.61 ± 0.08; T = 30 0.74 ± 0.15, Two-way ANOVA Genotype: F(1, 40) = 2.01, *p* = 0.16; Time: F(3, 40) = 0.16, *p* = 0.93; Interaction: F(3, 40) = 1.13, *p* = 0.35). **(B)** Representative immunoblots and quantification of phospho-T19 levels following treatment with vehicle (DMSO) or the Pin1 inhibitor juglone over a 120-min time course (0–120 min). Normalized intensity (AU; baseline = 1.0 at 0 min) was measured at 30, 60, and 120 min in DMSO (control) and juglone-treated samples across independent experiments (*n* = 9 per condition). Data are presented as mean ± SD. DMSO-treated samples showed mean intensities of 0.88 ± 0.11 (30 min), 1.10 ± 0.19 (60 min), and 0.65 ± 0.10 (120 min), while juglone-treated samples exhibited 0.74 ± 0.17, 0.97 ± 0.28, and 0.70 ± 0.56 at the respective time points. A two-way repeated-measures ANOVA (treatment × time) revealed no significant main effect of treatment and no treatment × time interaction (*p* > 0.05), although a significant effect of time was observed (*p* < 0.05). Post hoc comparisons (Tukey’s test) did not detect significant differences between DMSO and juglone at any individual time point. Notably, juglone-treated samples displayed increased variability at later time points, particularly at 120 min. **(C)** Western blot and accompanying graph showing the increase in T19 phosphorylation in the presence of GSK3ß and the decrease in the signal when Pin1 is overexpressed [(−) GSK3β 1.02 ± 0.02; (+) GSK3β 2.20 ± 0.34; GSK3β + Pin1 0.37 ± 0.10, One-way ANOVA F(2, 24), *p* < 0.001].

Pin1 regulates the stability of many proteins by altering their phosphorylation state (Hamdane et al., 2006; Kimura et al., 2013; Raghuram et al., 2013; Saxena et al., 2010; Tong et al., 2015; Zhou et al., 2000). In addition, the phosphodeficient forms of PSD-95 did not demonstrate measurable levels of ubiquitination, suggesting that phosphorylation of at least S25 promotes PSD-95 ubiquitination and T19 prevents its ubiquitination. To see if Pin1 and its isomerase function alters the levels of phospho-T19 acutely, HEK293T cells were transfected with PSD-95 and Pin1 or the K63A isomerization deficient mutant of Pin1. These cells were also treated with 10 μM cantharidin—a PP1/PP2A inhibitor (Delgado et al., 2007)—for the indicated period. Cell overexpressing Pin1 show a modest increase in the levels of phospho-T19 by the 10-min mark while cells overexpressing the K63A mutant show a decrease (Figure 3A, Wt Pin1: T = 0, 1.00 ± 0.08; T = 10, 1.31 ± 0.13; T = 20, 1.17 ± 0.13; T = 30, 1.06 ± 0.10 vs. K63A: T = 0, 1.00 ± 0.17; T = 10, 0.70 ± 0.08; T = 20, 0.61 ± 0.08; T = 30 0.74 ± 0.15); however, this difference did not reach statistical significance (Two-way ANOVA Genotype: F(1, 40) = 2.01, *p* = 0.16; Time: F(3, 40) = 0.16, *p* = 0.93; Interaction: F(3, 40) = 1.13, *p* = 0.35).

To maximize the levels of GSK3 activity, we starved cells for 18 h before harvesting. Despite this manipulation being well known for the increase in GSK3β activity (Esa and Tor, 2012; Xia et al., 2022), it failed to change the level of T19 phosphorylation significantly (Supplementary Figure 1A, fed 1.00 ± 0.20; starved 1.27 ± 0.20, *n* = 7, un-paired t-test *p* = 0.054). The manipulation did increase the proteolytic processing of PSD-95 evident as an increase in the amount of a band recognized by the anti-PSD-95 antibody running at approximately 37 kDa (Supplementary Figure 1B, fed 1.00 ± 0.25; starve 1.97 ± 0.27, *n* = 7, un-paired t-test *p* = 0.0005), suggesting that cell starvation leads to PSD-95 degradation, resulting in fragments of PSD-95 at approximately 37 kDa. Taking together, we were unable to observe changes in T19 phosphorylation in the matter of minutes to hours, suggesting that T19 is under tight control.

To further evaluate the role of Pin1 in the regulation of T19 phosphorylation or dephosphorylation, we inhibited Pin1 with 20 μM Juglone, a Pin1 inhibitor, for 30, 60 or 120 min and measure the phosphorylation state of T19 in cell lysates via western blot. In agreement with the results in Figure 3A, the levels of phospho-T19 were undistinguishable from control levels at all time points (Figure 3B, DMSO: T = 0 1.00 ± 0.00, T = 30 0.88 ± 0.11, T = 60 1.10 ± 0.19, T = 120 0.65 ± 0.10; Juglone: T = 0 1.00 ± 0.00, T = 30 0.74 ± 0.17, T = 60 0.97 ± 0.28, T = 120 0.70 ± 0.56). Over a period of two-hours we observed no significant differences in the levels of phospho-T19 when Pin1 is inhibited by Juglone (Two-way ANOVA Drug Treatment: F(1, 64) = 0.69, *p* = 0.41; Time: F(3, 64) = 0.31, *p* = 0.82; Interaction: F(3, 64) = 0.71, *p* = 0.55).

Since Pin1 manipulations do not change the levels of pT19 in PSD-95 in a short period of time, we evaluated if Pin1 altered the levels of pT19 in a slower time scale. To test this, cells were transfected with plasmids encoding PSD-95, GSK3β, and Pin1 and measure the phosphorylation state of T19 in cell lysates. GSK3β overexpression increases the level of PSD-95 phosphorylation, while Pin1 overexpression led to a one and half fold decrease in the phosphorylation of T19 in PSD-95 (Figure 3C, (−) GSK3β 1.02 ± 0.02; (+) GSK3β 2.20 ± 0.34; GSK3β + Pin1 0.37 ± 0.10, One-way ANOVA F(2, 24) = 6.08, *p* = 0.015). The reduction in T19 phosphorylation is disproportionate to the modest change in GSK3β levels. Taken together, we observed Pin1 negatively regulates the level of T19 phosphorylation on a slower timescale (days) but not in the minute to hour time domain. This slow effect on PSD-95 phosphorylation impacts the capacity of PSD-95 to become ubiquitinated.

### Pin1 opposes the GSK3β action on excitatory synaptic strength

**Figure 4:**
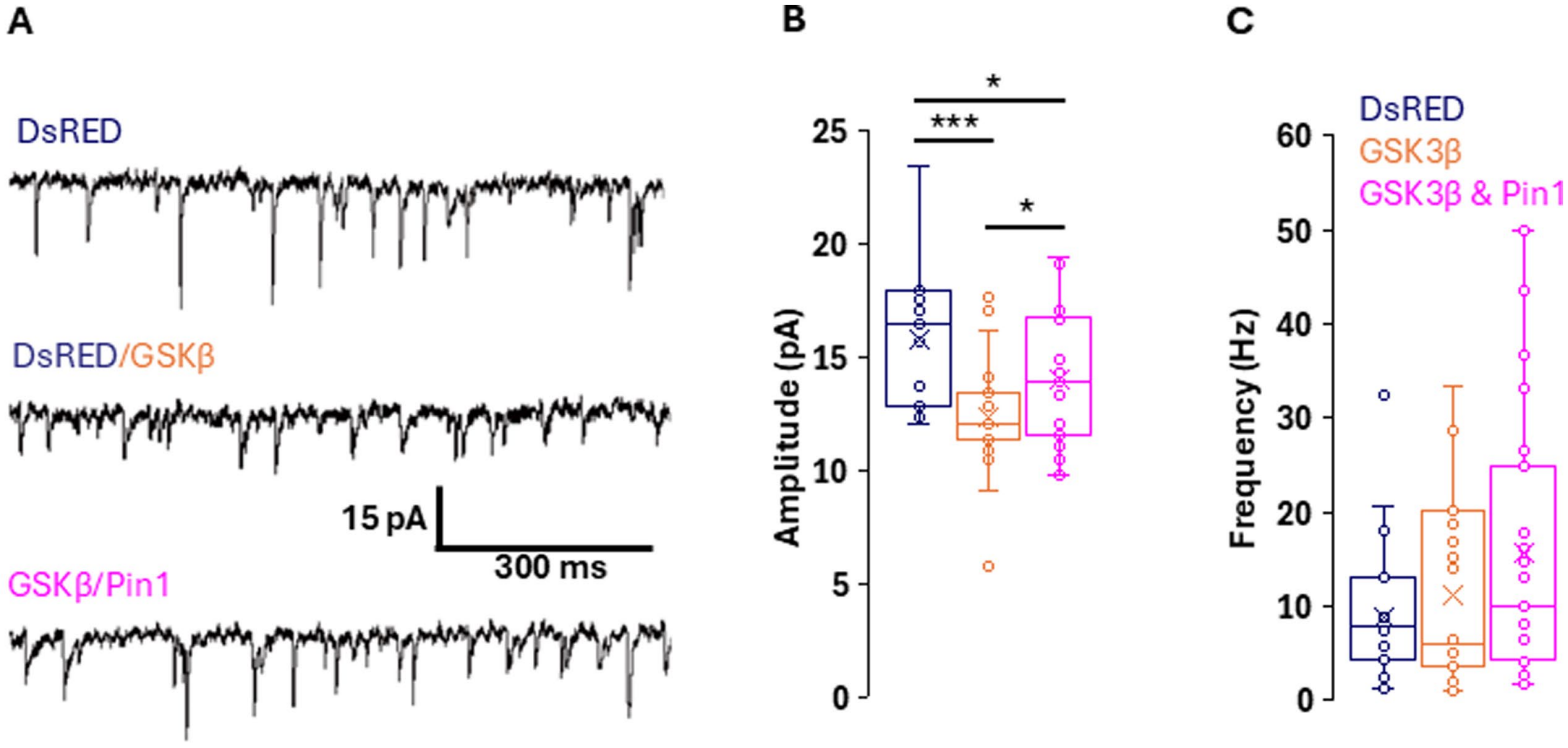
Pin1 rescues the depression of synaptic transmission induced by GSK3β overexpression. **(A)** mEPSC traces from the neurons expressing the various conditions. **(B)** Box plots showing the statistically significant decrease in the amplitude of the mEPSCs in cells overexpressing GSK3β. Pin1 reverts the depressive effects induced by GSK3β (DsRED 15.47 ± 0.62 pA, *n* = 27; GSKβ 12.56 ± 0.62 pA, *n* = 31; GSKβ + Pin1 13.61 ± 0.63 pA, *n* = 35, One-way ANOVA with Turkey’s multiple comparison test, F(2, 90) = 11.47, *p* = 0.000037) **(C)** Overexpression of GSKβ and GSKβ with Pin1 does not alter the frequency of the mEPSCs.

The function of GSK3β and its paralog GSK3α has been studied in relation to their role in maintaining excitatory synaptic transmission in the hippocampus and LTD (Bradley et al., 2012; Draffin et al., 2021; Peineau et al., 2007), the role of Pin1 in opposing GSK3 action on LTD is less well understood. To assess the role of GSK3B and Pin1 in the regulation of excitatory synaptic transmission, we measure miniature excitatory postsynaptic currents (mEPSCs) as the effects of GSK3 on AMPAR/NMDARs have been examined previously by Draffin et al. (2021). Similarly to their results, neurons overexpressing GSK3β, demonstrated a significant reduction in the amplitude of the mEPSCs (Figures 4A–C, DsRED 15.47 ± 0.62 pA, *n* = 27; GSKβ 12.56 ± 0.62 pA, *n* = 31; GSKβ + Pin1 13.61 ± 0.63 pA, *n* = 35), One-way ANOVA with Turkey’s multiple comparison test, F(2, 90) = 11.47, *p* = 0.000037), similar to the effects observed by Draffin et al. (2021). This effect was partially rescued by the overexpression of Pin1 (Figure 4B, A post-hoc test using Tukey HSD revealed significant differences between DsRED and GSK3β (*p* = 0.000019), as well as significant differences between DsRED and GSK3β + Pin1 (*p* = 0.04079), and a significant between GSK3β and GSK3β + Pin1 (*p* = 0.0333). No effect on the frequency of the mEPSCs was observed (Figure 4C). While there was no change in the AMPAR/NMDA ratio in cells where Pin1 protein levels were altered (Supplementary Figures 2B,C, control 0.95 ± 0.16, *n* = 7; Pin1 0.94 ± 0.39; Pin1 shRNA 1.63 ± 0.41, *n* = 7) and there was no change in the amplitude or the frequency of the mEPSCs in cells where Pin1 was overexpressed or knockdown (Supplementary Figures 2D–F, amplitude control, *n* = 54, 20.33 ± 0.49 pA; Pin1, *n* = 27, 20.15 ± 0.62 pA; Pin1 shNRA, *n* = 53, 19.83 ± 0.45 pA; mEPSC frequency control, 18.48 ± 2.28 Hz; Pin1 19.20 ± 3.59 Hz; Pin1 shRNA 13.59 ± 1.90 Hz). Pin1 counteracts the reduction in synaptic transmission caused by GSK3β overexpression, an effect that may be linked to Pin1-dependent regulation of PSD-95 phosphorylation and ubiquitination.

**Figure 5:**
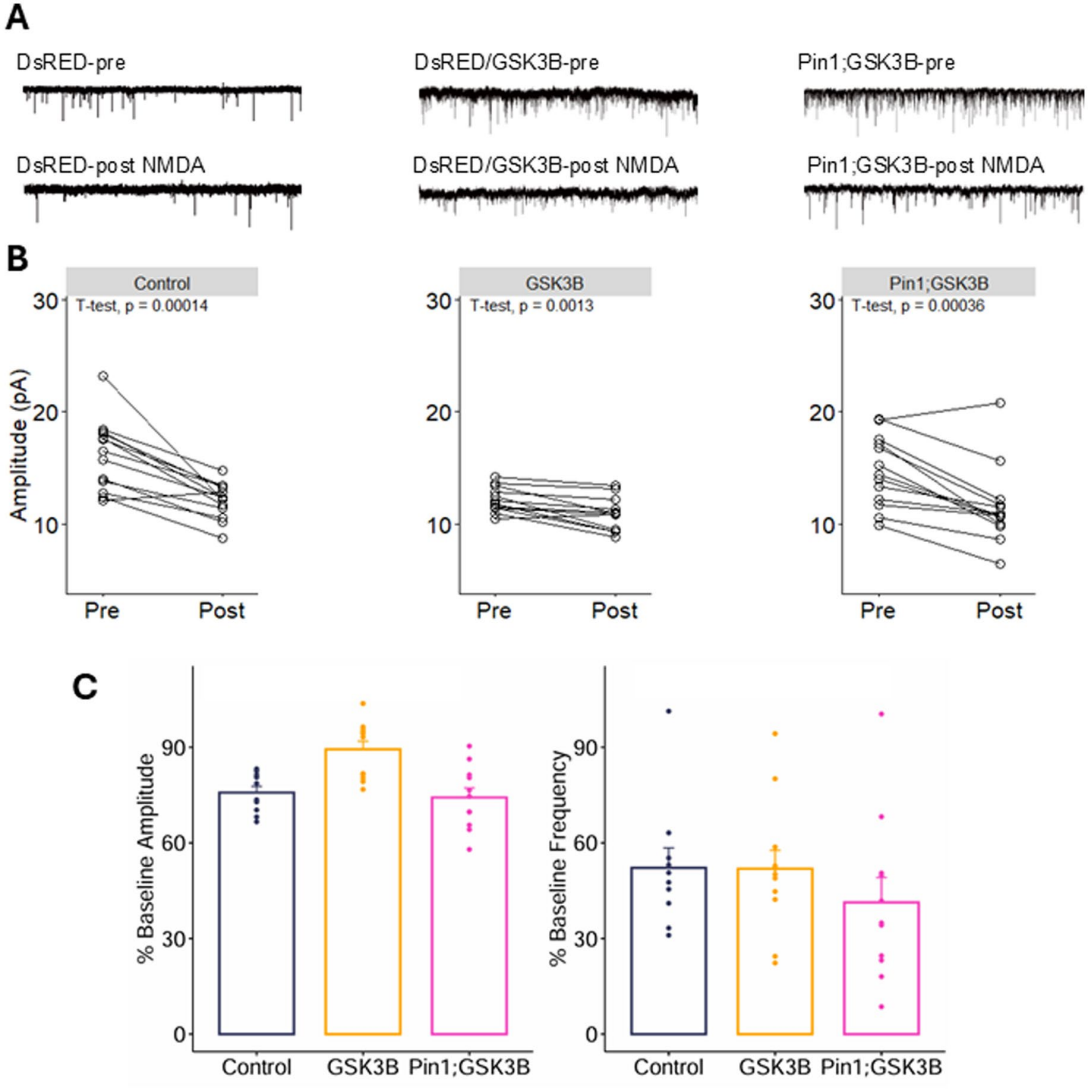
Pin1 rescues the suppression of NMDA-LTD induced by GSK3β overexpression. (Top) mEPSC traces recorded from control neurons, neurons overexpressing GSK3β, or GSK3β + Pin1. (Middle) Average mEPSC amplitude before (pre) the application of 20 μM NMDA for 5 min and after the application (post). The value of the paired *t*-test is shown above for all conditions, Control 0.76 ± 1.97, *n* = 10, *p* = 0.00014; GSK3β 0.89.35 ± 0.03, *n* = 12, *p* = 0.0013; GSK3β + Pin1 0.74 ± 0.03, *n* = 11, *p* = 0.00036. (Bottom) Average LTD expressed in control conditions vs. neurons overexpressing GSK3β or GSK3β + Pin1. LTD was induced in all conditions with a statistically significant reduction in neurons overexpressing GSK3β alone, Control 76% ± 1.97, *n* = 10; GSK3β 89.35% ± 0.03, *n* = 12; GSK3β + Pin1 74 ± 0.03%, *n* = 11, One-way ANOVA, F(2, 30) = 0.00027, Turkey multiple comparison of means, Control vs. GSK3β *p* = 0.0023, Control vs. GSK3β + Pin1 *p* = 0.91, GSK3β vs. GSK3β + Pin1 *p* = 0.00055. No change in the frequency was observed.

Lastly, we evaluated the interplay between GSK3B and Pin1 in the regulation of NMDAR-LTD. Chem LTD was induced chemically using a modification to the original protocol of Beattie et al. (2000) and Lee et al. (1998) as in Delgado et al. (2007). Instead of recording evoke responses, we recorded mEPSC amplitude before and after the bath application of 20 μM NMDA for 5 min in a modified ACSF solution containing 4 mM Ca^2+^ and 1.2 mM Mg^2+^, as per Delgado et al. (2007). This protocol induced reliable LTD in our experimental conditions (Supplementary Figure 3, mEPSC size pre 21.8 pA ± 3.40 vs. 15.46 ± 1.74 pA, *n* = 8, paired t-test *p* = 0.023). Manipulating the levels of Pin1 failed to alter the levels of NMDAR-LTD from control levels (Supplementary Figure 4, Control 0.74 ± 0.041, *n* = 12; *p* = 0.0003; Pin1 0.66 ± 0.03, *n* = 15, *p* = 2.8E-6; Pin1 shRNA 0.78 ± 0.03, *p* = 0.0004, One-way ANOVA *p* = 0.2). In all cells LTD was observed (Figures 5A,C, paired t-test, Control 0.76 ± 1.97, *n* = 10, *p* = 0.00014; GSK3*β* 0.89.35 ± 0.03, *n* = 12, *p* = 0.0013; GSK3β + Pin1 0.74 ± 0.03, *n* = 11, *p* = 0.00036); however, in neurons overexpressing GSK3β there was a statistically significant block in the levels of LTD that was rescued by the addition of Pin1 (Figures 5A,C, Control 76% ± 1.97, *n* = 10; GSK3β 89.35% ± 0.03, *n* = 12; GSK3β + Pin1 74% ± 0.03, *n* = 11, One-way ANOVA, F(2, 30) = 0.00027, Turkey multiple comparison of means, Control vs. GSK3β *p* = 0.0023, Control vs. GSK3β + Pin1 *p* = 0.91, GSK3β vs. GSK3β + Pin1 *p* = 0.00055). Taken together, these results suggest that Pin1 opposes the action of GSK3β on posttranslational modifications required for the establishment of baseline synaptic transmission and for the induction of NMDAR-LTD.

## Discussion

In this study, we investigated the relationship between proline-directed phosphorylation, cis–trans isomerization of PSD-95, and PSD-95 ubiquitination during NMDAR-dependent LTD. Strong prior evidence supported a mechanistic interplay between GSK3 activity and Pin1-mediated regulation of PSD-95 in the control of synaptic depression (Bianchetta et al., 2011; Delgado, 2020; Delgado et al., 2020; Draffin et al., 2021; Nelson et al., 2013). Our findings indicate that manipulations that alter phosphorylation at T19 are associated with corresponding changes in PSD-95 ubiquitination, while revealing that GSK3β binding to PSD-95 plays a more prominent role in regulating ubiquitination when T19A is mutated to alanine, suggesting that alternative sites may also be recognizable by GSK3β. In contrast, Pin1 acts in opposition to GSK3β as a negative regulator of T19 phosphorylation, resulting in reduced PSD-95 ubiquitination. Consistent with these biochemical observations, GSK3β and Pin1 exert opposing effects on baseline synaptic transmission and NMDAR-dependent LTD. Together, these findings refine current molecular models of LTD and indicate that regulation of PSD-95 by GSK3β and Pin1 extends beyond a simple linear pathway involving direct phosphorylation of T19.

Previous work has demonstrated a positive correlation between phosphorylation of the PSD-95 N-terminus and NMDAR-dependent LTD (Bianchetta et al., 2011; Nelson et al., 2013), supporting a cooperative regulatory mechanism involving both GSK3β and CDK5. Inhibition of CDK5 through genetic deletion of its activator p35 was shown to increase poly- and mono-ubiquitination of PSD-95, leading to enhanced association of AMPARs with the endocytic machinery following NMDA application (Bianchetta et al., 2011). This manipulation was also previously reported to alter phosphorylation of PSD-95 at serine 25 (S25) (Morabito et al., 2004), implicating S25 as a potential regulatory signal linking phosphorylation to ubiquitination. Consistent with prior reports, we observed similar ubiquitinated PSD-95 species, with prominent bands migrating at approximately 130 kDa and 105 kDa. However, our data further indicates that phosphorylation of PSD-95 at S25 is required for efficient ubiquitination, whereas phosphorylation at T19 acts as an inhibitory modulatory site (Figure 2). Notably, overexpression of GSK3β further enhanced PSD-95 ubiquitination, supporting a model in which GSK3β regulates ubiquitination through protein–protein interactions rather than direct phosphorylation of T19. This mode of regulation is consistent with established paradigms in which GSK3β participates in ubiquitin-dependent control of substrates such as β-catenin (Liu et al., 1999; MacDonald et al., 2009). Together, these findings suggest that PSD-95 ubiquitination is coordinately regulated by CDK5 and GSK3β, but through distinct and non-linear mechanisms that extend beyond simple kinase–substrate relationships.

Pin1 has been shown to regulate the ubiquitination of multiple protein targets through its ability to recognize phosphorylated Ser/Thr-Pro motifs and modulate substrate conformation (Krishnan et al., 2012; Lonati et al., 2014; Liou et al., 2011); however, its role in the ubiquitination of synaptic proteins has not been directly examined. Here, we show that Pin1 expression leads to a robust reduction in PSD-95 ubiquitination (Figure 2), consistent with prior observations of Pin1-dependent regulation of p53 stability (Siepe and Jentsch, 2009). Mapping experiments indicate that this effect is mediated through phosphorylation sites in the N-terminus of PSD-95, specifically T19 and S25 (Figures 2B–D), rather than phosphorylation sites located within the hinge domain (Antonelli et al., 2016; Coba et al., 2009). These findings support a model in which Pin1 associates with PSD-95 via pS25 and modulates the phosphorylation state of T19 through a conformational mechanism (Figure 3C). Notably, this process appears to occur on a relatively slow timescale, as acute inhibition of Pin1 or protein phosphatases did not produce rapid changes in T19 phosphorylation (Figures 3A,B). Together, these results suggest that Pin1 functions as a molecular timer that regulates T19 phosphorylation over longer timescales, positioning Pin1 as a key modulator of PSD-95 stability during synaptic plasticity.

At the synaptic level, overexpression of GSK3β reduced mEPSC amplitude and significantly attenuated NMDAR-dependent LTD (Figure 4), directly demonstrating a functional impact of GSK3β on synaptic depression. In contrast, Pin1 overexpression restored both baseline synaptic transmission (Figure 4) and LTD to control levels in the presence of elevated GSK3β (Figure 5). Based on the biochemical effects of Pin1 on T19 phosphorylation, one might anticipate that Pin1 overexpression would suppress LTD; however, this was not observed. Instead, consistent with the biochemical findings, Pin1 antagonized the effects of GSK3β on T19 phosphorylation, PSD-95 ubiquitination, and the suppression of synaptic transmission and LTD. Together, these results identify an opposing regulatory relationship between GSK3β and Pin1 that directly governs the stability of PSD-95 and the expression of NMDAR-dependent LTD at excitatory synapses. Notably, both GSK3β (and GSK3α) and Pin1 are present at relatively low levels at excitatory synapses (Alkaas et al., 2025), suggesting that the balance between these enzymes—rather than their absolute abundance—may be a key determinant of PSD-95-dependent synaptic plasticity under specific physiological conditions.

While our findings establish a functional interplay between GSK3β and Pin1 in regulating PSD-95 ubiquitination and synaptic plasticity, several mechanistic aspects remain unresolved. Although GSK3β association with PSD-95 promotes ubiquitination, our data suggest that this effect likely requires GSK3β kinase activity to regulate the phosphorylation and ubiquitination of additional components within the associated protein complex, rather than direct phosphorylation of PSD-95 at T19. In addition, we notice a reduction in PSD-95 levels in lysates expressing GSK3β in the T19A mutant, thus suggesting that direct phosphorylation of GSK3 enzyme is not needed for the effect. We were unable to identify the specific ubiquitin ligase responsible for PSD-95 ubiquitination or to definitively determine the lysine linkage involved, although prior work has implicated MDM2 and K63-linked ubiquitination of PSD-95 (Bianchetta et al., 2011; Colledge et al., 2003; Ma et al., 2017). In addition, while GSK3β and Pin1 exert opposing effects on synaptic transmission and LTD, we cannot exclude the possibility that these functional effects are mediated through phosphorylation sites or mechanisms beyond T19. Although our study focused on GSK3β, recent work suggests that GSK3α plays a dominant role in the induction of NMDAR-dependent LTD in CA1 neurons. Our findings do not exclude isoform-specific contributions to LTD signaling and instead indicate that GSK3 phosphorylation (as both isoforms recognize the same sites) may regulate synaptic plasticity through modulation of PSD-95 stability and associated protein complexes. Future studies will be required to determine whether GSK3α engages similar regulatory mechanisms at the level of postsynaptic scaffolds. These constraints define important boundaries for the present study and highlight key directions for future mechanistic dissection.

This study identifies a previously unappreciated regulatory pathway governing PSD-95 phosphorylation, ubiquitination, and stability that integrates the opposing actions of GSK3β, Pin1, and protein phosphatases. We propose a model in which basal CDK5 activity promotes phosphorylation of PSD-95 at S25, enabling association of GSK3β and recruitment of ubiquitin ligase complexes to PSD-95. This phosphorylation-dependent association is proposed to place PSD-95 in a conformationally permissive state for ubiquitination, whereas Pin1 binding acts to reverse this state by modulating N-terminal conformation and facilitating dephosphorylation. In the absence of Pin1 activity, sustained GSK3β-dependent signaling favors PSD-95 ubiquitination and synaptic depression, whereas Pin1 counteracts this process to stabilize PSD-95 and restore synaptic strength. Through this opposing regulatory balance, Pin1 emerges as a critical molecular gate that determines the capacity of excitatory synapses to maintain baseline transmission and to express NMDAR-dependent LTD.

## Data Availability

The original contributions presented in the study are included in the article/Supplementary material, further inquiries can be directed to the corresponding author.

## Supplementary material

SUPPLEMENTARY FIGURE 1 Cell starvation mediates PSD-95 degradation. **(A)** HEK 293T cells were either fed or starved. In fed conditions, the media was replaced 16-hours post transfection, in starved conditions the media was not replaced. Representative immunoblots and accompanying graph demonstrate no effect from the starvation manipulation on PSD-95 phosphorylation. Fed 1.00 ± 0.00; Starved 1.53 ± 0.30, *n* = 6, *p* = 0.145, un-paired t-test. **(B)** Representative immunoblots and accompanying graph show significantly higher PSD-95 signal at 36 kDa in the starved condition in comparison to the fed condition. Fed 1.00 ± 0.00; Starved 2.69 ± 0.50, *n* = 6, **p* = 0.02, unpaired t-test.

SUPPLEMENTARY FIGURE 2 Influence of Pin1 protein levels on baseline synaptic transmission. **(A)** Representative DIC imaged as an example of a recording cell. **(B)** Representative evoked AMPAR and NMDAR responses in varying experimental groups. **(C)** Quantification of the AMPAR/NMDAR ratio for each experimental group. **(D)** Representative mEPSC traces for the different groups. **(E)** Comparison of cumulative probability of EPSP amplitude and inter-event interval among control, Pin1 overexpression, and Pin1 knockdown experimental groups. **(F)** Average mEPSC amplitude and frequency showing a lack of alteration in the basic electrophysiological properties of the excitatory synapse. One-way ANOVA *p* = 0.14 and 0.23, respectively. Experiments in which series resistance increased by more than 100% were excluded from analysis.

SUPPLEMENTARY FIGURE 3 NMDAR-LTD under the conditions of 20uM NMDA and 4mM Ca2+ for 5 minutes. **(A)** Comparison of mEPSC traces before and after NMDAR-LTD treatment, depicting a decrease in the frequency and amplitude of the traces. **(B)** Changes in mEPSC amplitude (pA) after being subject to 5 minutes of NMDAR-LTD treatment, depicting an overall decrease in mEPSC amplitude.

SUPPLEMENTARY FIGURE 4 **(A)** mEPSC amplitude pre- and post-NMDA-LTD. Coverslips were treated with 20 uM NMDA and 4 mM CaCl2 for 5 minutes. Post-treatment amplitude was 15-20 minutes post. Paired t-tests: Control *p* = 0.0003; Pin1 *p* = 2.8E-6; shRNA *p* = 0.0004. **(B)** Representative traces from baseline and post NMDA-LTD. **(C)** Post-NMDA-LTD change in mEPSC amplitude (% of baseline). One-way ANOVA *p* = 0.2. **(D)** Post-NMDA-LTD change in mEPSC frequency (% of baseline). One-way ANOVA *p* = 0.49. Experiments in which series resistance increased by more than 100% were excluded from analysis. Control *n* = 10, Pin1 *n* = 10, shRNA *n* = 10 cells.
