# Supplementary figures and images for: Proline-directed phosphorylation and prolyl isomerization oppose each other to regulate PSD-95 ubiquitination and excitatory synaptic plasticity
Source: Front Mol Neurosci. 2026 Jun 16;19:1777680. doi: 10.3389/fnmol.2026.1777680 (PMC13314775; doi:10.3389/fnmol.2026.1777680)

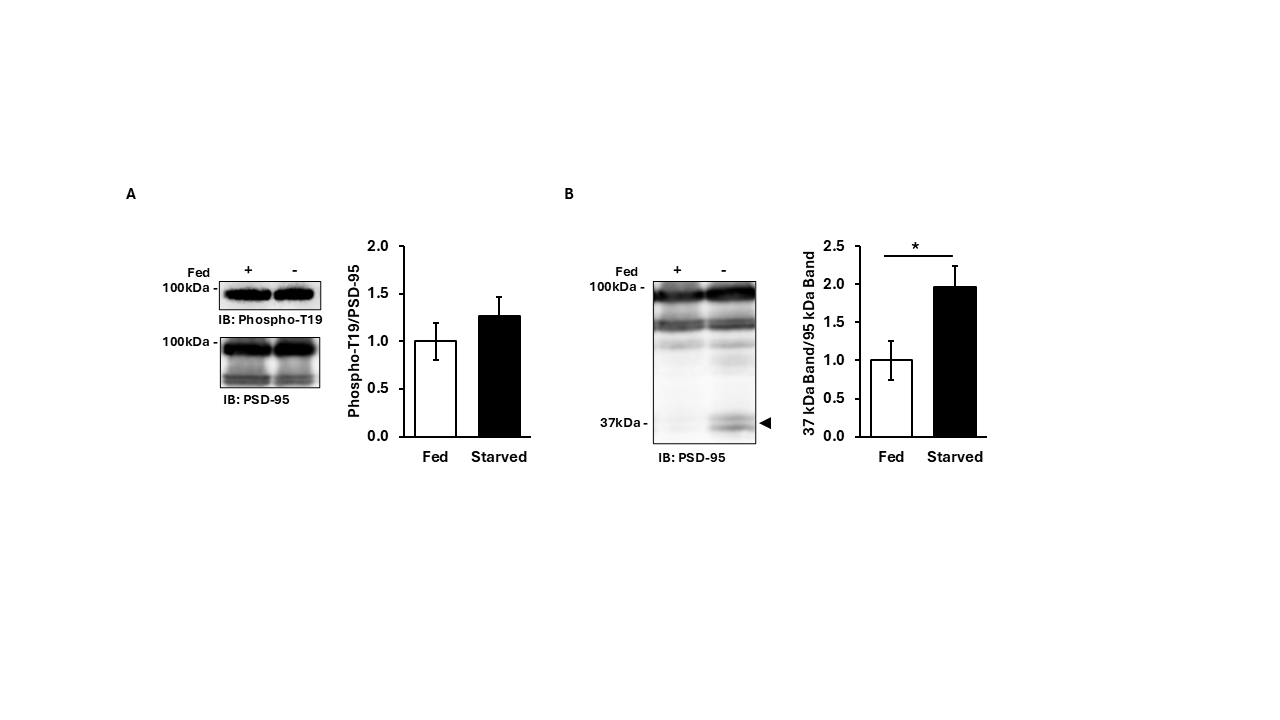

Supplement: SUPPLEMENTARY FIGURE 1 — Cell starvation mediates PSD-95 degradation. (A) HEK 293T cells were either fed or starved. In fed conditions, the media was replaced 16-hours post transfection, in starved conditions the media was not replaced. Representative immunoblots and accompanying graph demonstrate no effect from the starvation manipulation on PSD-95 phosphorylation. Fed 1.00 ± 0.00; Starved 1.53 ± 0.30, n = 6, p = 0.145, un-paired t-test. (B) Representative immunoblots and accompanying graph show significantly higher PSD-95 signal at 36 kDa in the starved condition in comparison to the fed condition. Fed 1.00 ± 0.00; Starved 2.69 ± 0.50, n = 6, *p = 0.02, unpaired t-test. [file Image_1.tif]

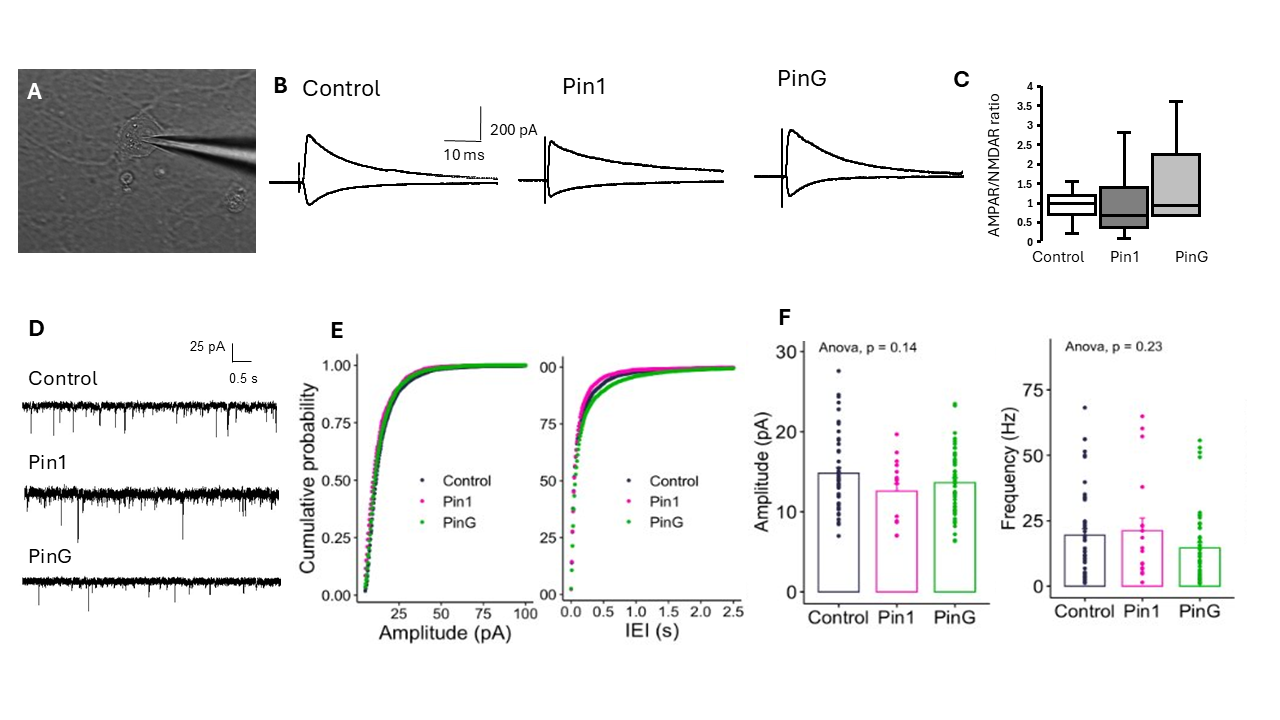

Supplement: SUPPLEMENTARY FIGURE 2 — Influence of Pin1 protein levels on baseline synaptic transmission. (A) Representative DIC imaged as an example of a recording cell. (B) Representative evoked AMPAR and NMDAR responses in varying experimental groups. (C) Quantification of the AMPAR/NMDAR ratio for each experimental group. (D) Representative mEPSC traces for the different groups. (E) Comparison of cumulative probability of EPSP amplitude and inter-event interval among control, Pin1 overexpression, and Pin1 knockdown experimental groups. (F) Average mEPSC amplitude and frequency showing a lack of alteration in the basic electrophysiological properties of the excitatory synapse. One-way ANOVA p = 0.14 and 0.23, respectively. Experiments in which series resistance increased by more than 100% were excluded from analysis. [file Image_2.tif]

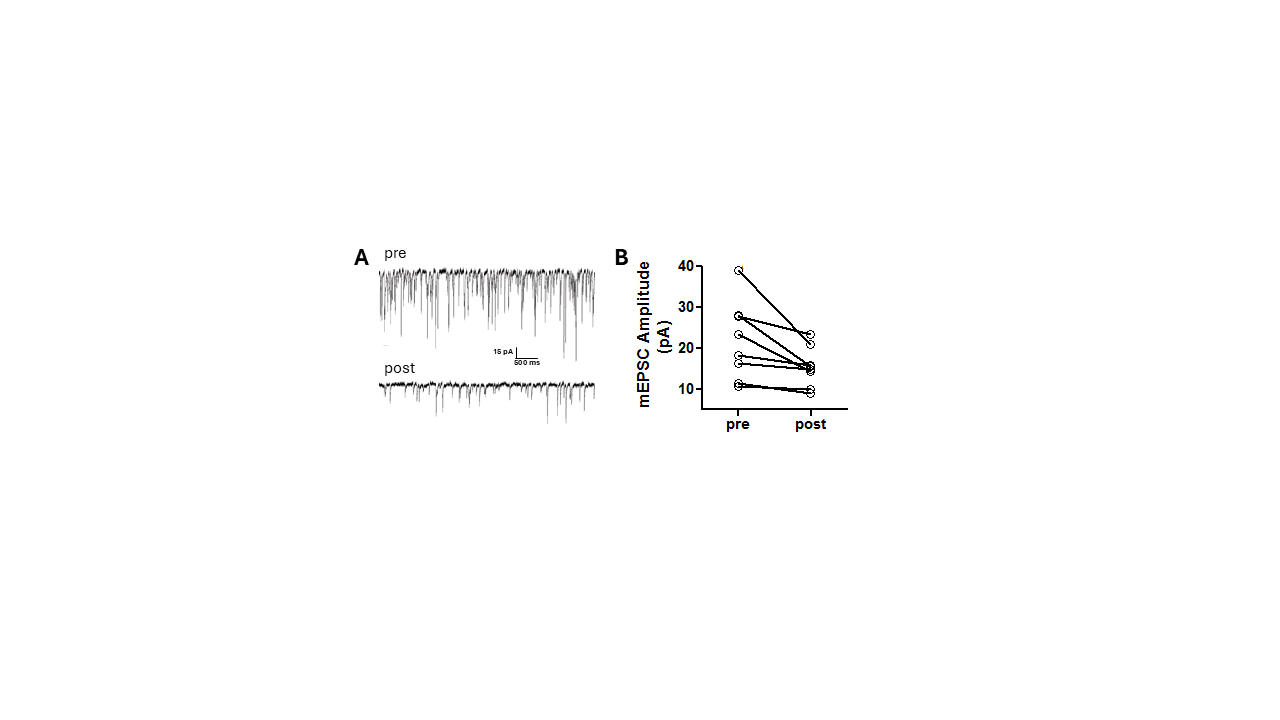

Supplement: SUPPLEMENTARY FIGURE 3 — NMDAR-LTD under the conditions of 20uM NMDA and 4mM Ca2+ for 5 minutes. (A) Comparison of mEPSC traces before and after NMDAR-LTD treatment, depicting a decrease in the frequency and amplitude of the traces. (B) Changes in mEPSC amplitude (pA) after being subject to 5 minutes of NMDAR-LTD treatment, depicting an overall decrease in mEPSC amplitude. [file Image_3.tif]

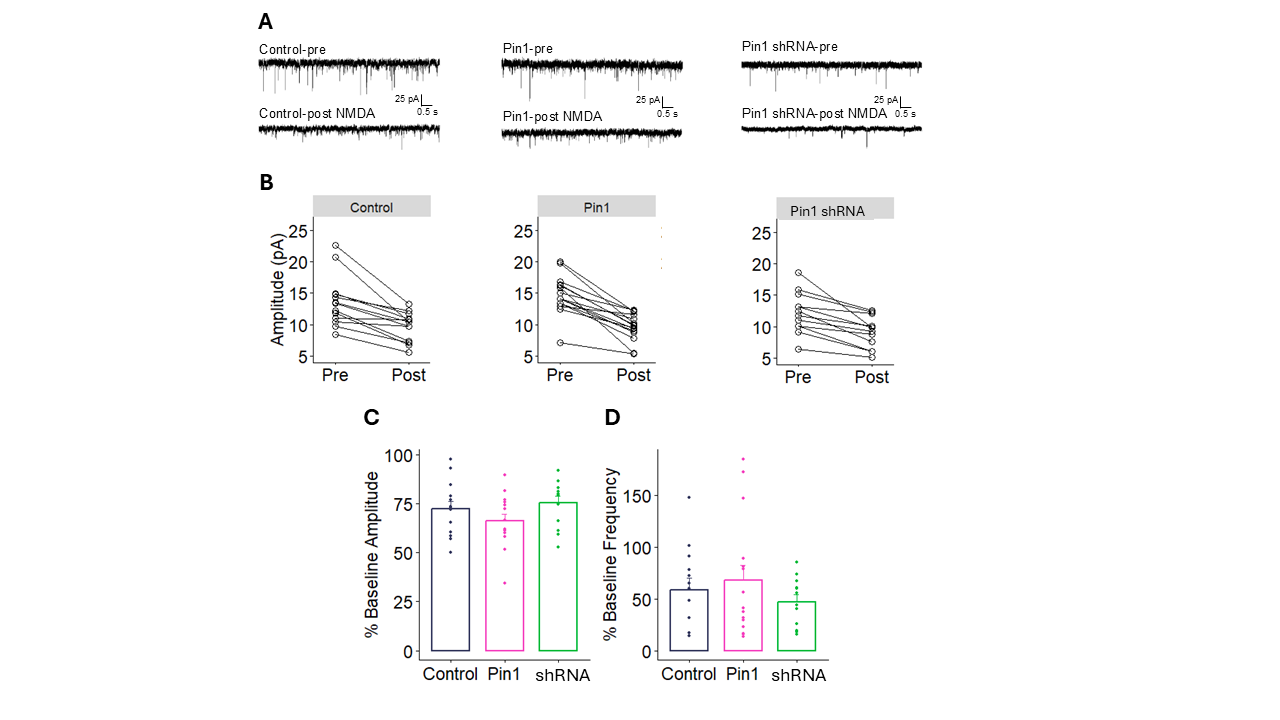

Supplement: SUPPLEMENTARY FIGURE 4 — (A) mEPSC amplitude pre- and post-NMDA-LTD. Coverslips were treated with 20 uM NMDA and 4 mM CaCl2 for 5 minutes. Post-treatment amplitude was 15-20 minutes post. Paired t-tests: Control p = 0.0003; Pin1 p = 2.8E-6; shRNA p = 0.0004. (B) Representative traces from baseline and post NMDA-LTD. (C) Post-NMDA-LTD change in mEPSC amplitude (% of baseline). One-way ANOVA p = 0.2. (D) Post-NMDA-LTD change in mEPSC frequency (% of baseline). One-way ANOVA p = 0.49. Experiments in which series resistance increased by more than 100% were excluded from analysis. Control n = 10, Pin1 n = 10, shRNA n = 10 cells. [file Image_4.tif]
